# Leveraging AlphaFold2 structural space exploration for generating drug target structures in structure-based virtual screening

**DOI:** 10.1016/j.bbrep.2025.102110

**Published:** 2025-07-11

**Authors:** Keisuke Uchikawa, Kairi Furui, Masahito Ohue

**Affiliations:** Department of Computer Science, School of Computing, Institute of Science Tokyo, 4259 G3-56 Nagatsuta-cho, Midori-ku, Yokohama, 226-8501, Kanagawa, Japan

**Keywords:** AlphaFold2, Structure-based virtual screening, Structure-based drug design, Protein structures, Conformational changes

## Abstract

Computational virtual screening (VS) plays a vital role in early-stage drug discovery by enabling the efficient selection of candidate compounds and reducing associated costs. However, the absence of experimentally determined three-dimensional protein structures often limits the applicability of structure-based VS. Advances in protein structure prediction, notably AlphaFold2, have begun to address this gap. Yet, studies indicate that direct use of AlphaFold2-predicted structures often leads to suboptimal VS performance—likely because these structures fail to capture ligand-induced conformational changes (apo-to-holo transitions). To overcome this, we propose an approach that explores and modifies the structural space of AlphaFold2 predictions to generate conformations more amenable to VS. Our method deliberately alters the multiple sequence alignment (MSA) by introducing alanine mutations at key residues in the ligand-binding site, thereby inducing significant conformational shifts. The exploration process is guided by iterative ligand docking simulations, with mutation strategies optimized either by a genetic algorithm or via random search. Our evaluation shows that when sufficient active compounds are available, the genetic algorithm significantly enhances VS accuracy. In contrast, with limited active compound data, a random search strategy proves more effective. Moreover, our approach is particularly promising for targets that yield poor screening results when using experimentally determined structures from the PDB. Overall, these findings underscore the practical utility of modified AlphaFold2-derived structures in VS and expand the potential of computationally predicted protein models in drug discovery.

## Highlights

• Modified protein alignments yield drug-friendly AlphaFold2 structures.
• Genetic search optimizes predicted structures for improved virtual screening.
• Enhanced screening performance seen for targets with poor PDB data.
• Method works for new targets and compares favorably with AlphaFold3.

## Graphical abstract

**Figure:**
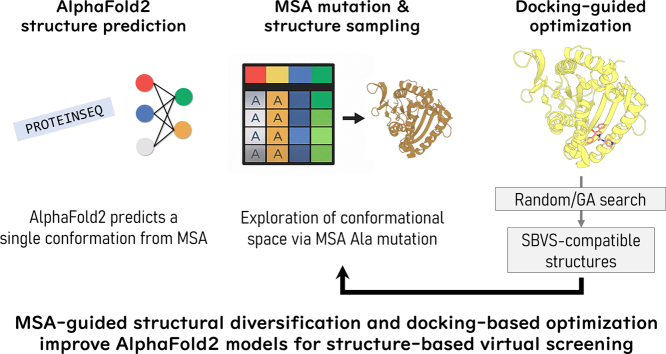

## 1 Introduction

Drug discovery is an expensive and time-consuming process, with estimated costs reaching several billion US dollars and timelines exceeding a decade [1], [2]. One of the major bottlenecks is the efficient identification of suitable drug candidates from the vast chemical space, estimated to contain between $10^{30}$ and $10^{60}$ molecules [3], [4]. Structure-based virtual screening (SBVS), which uses the 3D structure of target proteins to screen potential ligands, is a promising strategy to reduce development costs and timelines [5]. However, its accuracy and utility are highly dependent on the quality of the protein structure used. In particular, the lack of experimentally determined holo (ligand-bound) structures in the Protein Data Bank (PDB) often hinders the success of SBVS [6], [7], [8].

**Fig. 1:**
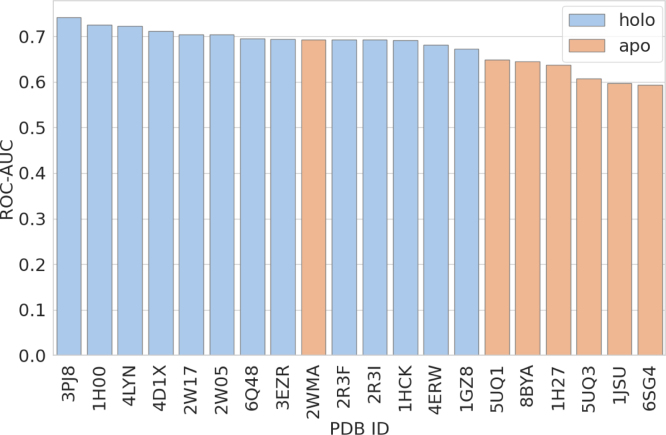
Variability in screening performance among crystal structures of CDK2. Docking simulations were performed using Uni-Dock [9] with the DUD-E dataset [10]. Performance was evaluated based on ROC–AUC for distinguishing actives from decoys.

As shown in Fig. 1, even for the same protein (CDK2), different crystal structures result in significantly different virtual screening outcomes. This highlights the critical role of structural selection in SBVS and motivates the need for alternative conformational exploration strategies.

To address this limitation, recent advances in protein structure prediction – especially the development of AlphaFold2 – have enabled highly accurate tertiary structure predictions from amino acid sequences [11]. These predictions are revolutionizing the field by providing access to structural information for previously uncharacterized proteins. However, several studies have shown that AlphaFold2-predicted structures perform similarly to apo (ligand-unbound) structures and worse than holo structures in SBVS tasks [12], [13]. Refinement strategies such as energy minimization, side-chain reorientation, and loop modeling have been proposed [14], but many rely on manual intervention or prior knowledge of holo structures, limiting their generalizability.

In this study, we propose a systematic approach to explore and optimize the conformational space of AlphaFold2-predicted protein structures for enhanced SBVS performance. Our method introduces alanine mutations at residues within the ligand-binding site in the multiple sequence alignment (MSA), which is known to influence AlphaFold2 predictions [15]. To guide the generation of SBVS-friendly conformations, we conduct iterative docking simulations using either a random search or a genetic algorithm to optimize the mutation pattern. The goal is to discover protein structures that more accurately distinguish active compounds from decoys, even when experimentally determined holo structures are unavailable.

The novelty of our work lies in combining MSA perturbation with score-guided structural optimization to generate diverse, SBVS- compatible conformations directly from AlphaFold2. Unlike prior methods that require manually curated refinements or holo structure templates, our approach is generalizable to novel proteins and scalable across different data availability conditions. We experimentally validate the method across multiple targets, including proteins outside AlphaFold2’s training set, and show that it improves SBVS performance compared to PDB structures and default AlphaFold2 models. Our results highlight the practical utility of this framework for structure-guided drug discovery and demonstrate the untapped potential of computationally generated structural ensembles.

## 2 Materials and methods

**Fig. 2:**
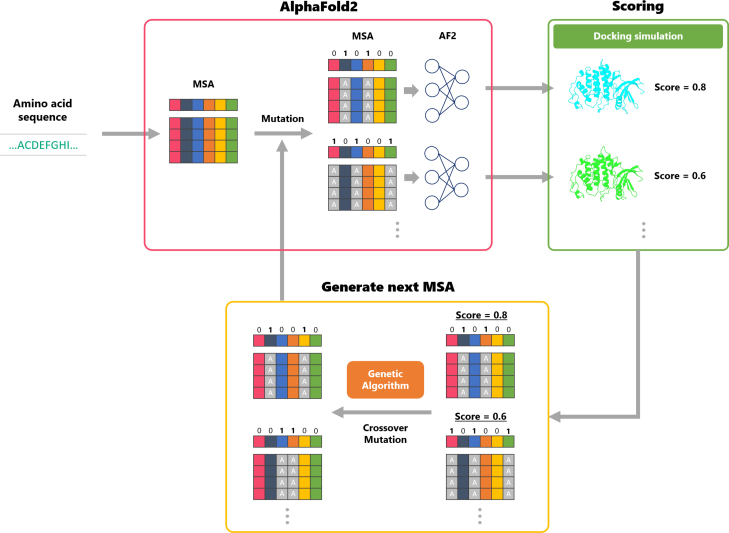
An overview of our approach using a genetic algorithm for exploration.

AlphaFold2 leverages information from multiple sequence alignments (MSAs) in its structure predictions, and numerous recent approaches have been proposed that manipulate MSAs to generate diverse structural states [15], [16], [17]. In this study, we focus on the approach by Stein et al. [15], which demonstrated that substituting residues with alanine along the MSA columns can alter inter-residue interactions and thereby produce different predicted structures. This result suggests that this approach may serve as a powerful means for obtaining diverse structure predictions. Building on these findings, we evaluate the impact of various alanine substitution patterns in the MSA on the predicted structures using docking simulation scores with exploration compounds. Based on the results, we explore for alanine substitution patterns that can generate predicted structures suitable for SBVS-structures that enable the accurate evaluation of novel compounds. As a score-based exploration approach, we implemented both random search and a genetic algorithm [18], [19], [20], and evaluated exploration using both a basic search strategy and an optimization strategy. The reason for investigating the genetic algorithm is that the combinations of alanine substitutions in the MSA are enormous, necessitating an efficient method to explore high-scoring configurations. We adopted the genetic algorithm because it can be applied to nonlinear problems with large solution spaces and, since each individual can be evaluated independently, it is well-suited for parallel processing [21]. These characteristics make it appropriate for the present problem, which is nonlinear and computationally intensive. Fig. 2 provides an overview of our approach using a genetic algorithm for exploration. First, using AlphaFold2, we generate a set of predicted structures based on MSAs with various alanine substitutions. Next, docking simulations with compounds for exploration are performed on these predicted structures, and each structure is scored. Based on these scoring results, operations such as crossover and mutation are applied to the alanine substitution patterns to generate the MSAs for the next generation. By repeating this process, we aim to generate predicted structures that achieve high screening performance even when evaluated with test compounds that were not used in exploration. In the case of using random search for exploration, the alanine substitution patterns are determined completely at random. In this study, we define “structures suitable for SBVS” as those that can classify active and inactive compounds more accurately in docking simulations.

### 2.1 Generating predicted structures with AlphaFold2

We used LocalColabFold [22] to generate predicted structures, as it allows us to specify certain parameters during structure prediction.studies have reported that by reducing both the number of MSAs and the number of recycling steps, one can induce conformational changes in the predicted structure [16]. Accordingly, we fixed the parameters so that LocalColabFold uses shallow MSAs and a minimal number of recycles. The detailed configuration is shown in Supporting Information S1. Input sequences for each target were obtained from UniProt [23]. Furthermore, in using the predicted structures, we trimmed regions with pLDDT <50 and removed parts that were deemed unreliable to prevent disordered regions from interfering with the binding site. The pLDDT score is a residue-level confidence metric output by the AlphaFold model: any residue with pLDDT <50 is considered to have low predictive reliability [11]. If the overall average pLDDT of a predicted structure was below 50, that predicted structure was not used.

### 2.2 Exploration of alanine substitution patterns in MSA

In this study, we explore alanine substitution patterns in the MSA that enable AlphaFold2 to generate structures more suitable for SBVS. Alanine substitution candidates are restricted to residues located within 8 Å of the ligand. This is because such residues are likely to be critical for protein–ligand interactions within the binding site, thus impacting the success or failure of docking. In this experiment, the binding site was treated as known and extracted from the holo PDB structure. Residues selected for alanine substitution were chosen based on the distance between the ligand in the holo PDB structure and the standard AlphaFold2 predicted structure of the UniProt input sequence. If any atom of a residue was within 8 Å of the ligand, the entire residue was considered to be within 8 Å. In our exploration approach, the presence or absence of an alanine substitution is represented as a 1/0 bit in a genetic representation. Concretely, for each column in the MSA generated by AlphaFold2, a bit value of 1 indicates that all residues in that column should be substituted with alanine (Fig. 2). Our substitution procedure follows the same approach as in the previous study [15]. Alanine was selected because it disrupts local interactions while exerting minimal influence on the overall protein fold. The mutation strategy is designed to induce localized perturbations near the binding site. Although such substitutions may interfere with native interactions (e.g., hydrogen bonds, hydrophobic packing), they facilitate the sampling of alternative conformations that are otherwise inaccessible. The detailed configuration is shown in Supporting Information S2.

### 2.3 Scoring method for predicted structures

To perform our exploration approach, we need a mechanism to score how suitable each predicted structure – obtained by mutating the MSA – is for SBVS. In this study, we adopted protein–ligand docking simulations for the scoring process.

#### 2.3.1 Docking simulation settings

For docking simulations, we used Uni-Dock [9], which improves efficiency through GPU usage compared to conventional AutoDock Vina [24], enabling fast and accurate docking. Given the need for docking simulations on a large number of predicted structures, Uni-Dock’s speed is a significant advantage from a computational cost perspective. We defined the docking grid by referring to experimental structure data from the PDB. Specifically, we used PyMOL [25] to extract the ligand’s central coordinates from the holo PDB structure and set the docking grid box accordingly. The grid size was set to 20 Å × 20 Å × 20 Å. Docking with Uni-Dock was performed with the scoring function set to “vina”, the search mode set to “balance”, and the random seed fixed at 1. We superimposed the predicted structure with the holo PDB structure such that the binding site residues within 8 Å of the ligand were aligned, ensuring that the docking center would match between them. For protein and ligand preprocessing, we used AutoDock Tools [26].

In this study, we focused on target proteins with well-defined ligand-binding sites, such as enzymes or established drug targets. The docking center was determined by superimposing the predicted structure onto the holo PDB structure provided by the DUD-E dataset and extracting the ligand centroid as the center of the docking box. This strategy assumes that at least one experimentally determined holo structure is available for the target. In cases where no such experimental structures exist, alternative methods for identifying the binding site are required. Our approach can be combined with structure-based binding site prediction tools such as Fpocket [27] or DynamicBind [28], enabling broader applicability to novel targets with unknown binding sites. We consider this an important direction for future work.

#### 2.3.2 Evaluation metrics for predicted structures

We calculated the ROC–AUC value [29] based on the ranking of ligands by their docking scores, taking into account their known active or inactive labels. This ROC–AUC value served as the score for each predicted structure, guiding the genetic algorithm’s operations (e.g., crossover, mutation). The ROC curve is a plot of the true positive rate against the false positive rate, and the AUC is the area under this curve. ROC–AUC values range from 0 to 1, with values closer to 1 indicating higher predictive accuracy. A value of 0.5 or below suggests classification performance equivalent to or worse than random guessing. In the SBVS context, a higher ROC–AUC value indicates that active compounds were more accurately distinguished from inactive compounds by the predicted structure (i.e., that the structure is more suitable for screening). We chose ROC–AUC because it is commonly used for evaluating screening performance and yields values in the interval [0, 1], making it convenient for use as a score in a genetic algorithm.

#### 2.3.3 Monitoring optimization behavior

**Fig. 3:**
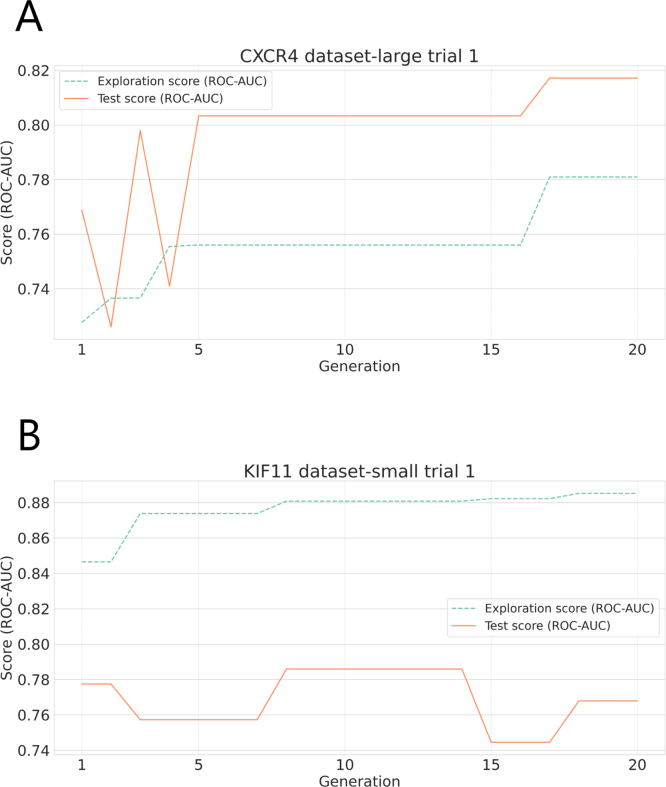
Examples of the transition of exploration in a genetic algorithm. A shows trial 1 results for CXCR4 (dataset-large). B shows trial 1 results for KIF11 (dataset-small). In both cases, exploration and evaluation were performed. The exploration score is the ROC–AUC from docking simulations using the exploration set of ligands, and the test score is the ROC–AUC from docking simulations using the test set of ligands.

To evaluate the progression of optimization during genetic algorithm exploration, we tracked the best-performing individual’s ROC–AUC score in each generation based on docking simulations with the exploration ligand set. These scores were plotted across generations to analyze convergence behavior. Representative examples are shown in Fig. 3.

### 2.4 Dataset

In this study, we selected both proteins that AlphaFold2 has already trained on and those that AlphaFold2 has not trained on as our targets. For targets trained on by AlphaFold2, we used the well-known DUD-E dataset [10], a benchmark dataset for SBVS that includes 102 proteins from diverse classes such as kinases, proteases, nuclear receptors, GPCRs, ion channels, and enzymes. Each target is associated with multiple active and inactive compounds, enabling evaluation of screening performance by measuring binary classification metrics. From this dataset, we selected CXCR4 [30] and KIF11 [31] from the Diverse Subset, as well as the canonical kinase CDK2 [32]. We chose CXCR4 because it yields the lowest screening performance and KIF11 because it yields the highest screening performance within the Diverse Subset [24]. This allowed us to thoroughly test whether our proposed method is effective. CDK2 is a typical kinase with a larger number of known active compounds in DUD-E relative to CXCR4 and KIF11, providing ample data for our experiments. To verify whether our approach can be applied to novel proteins not learned by AlphaFold2, we also selected targets that fall outside of AlphaFold2’s training data. The version of AlphaFold2 (LocalColabFold v1.5.5) used in our experiments was trained on PDB structures deposited up to April 30, 2018 [11]. Therefore, any protein deposited in the PDB after that date can be considered unknown to AlphaFold2. We searched for proteins satisfying the criteria that (1) they were not learned by AlphaFold2 or its close homologs, (2) a holo crystal structure is currently available, and (3) there is sufficient information on active compounds. Consequently, we selected ABHD6 (PDB ID: 7OTS [33]) and HIPK3 (PDB ID: 7O7J) [34] as targets. Their active compounds were obtained from ChEMBL [35], [36], where any compound with an IC_50_ < 10 nM was defined as active. We then generated decoy compounds for these active compounds using DUD-E Generate Decoys [10]. The detailed information is shown in Supporting Information S3.

### 2.5 Splitting the ligand data

In this study, the exploration uses an ROC–AUC score based on protein–ligand docking simulations with active and inactive compounds. To examine the effect of the number of known active compounds, we evaluate performance by varying the number of ligands used in the exploration under the following three conditions:

(dataset-large): two-thirds of the known active and inactive compounds were used for exploration, while the remaining one-third were reserved for testing. This setting aims to investigate exploration performance when sufficient ligand data are available. We performed 3-fold cross-validation, splitting the data by clustering based on Tanimoto distances.
(dataset-middle): 30 known active compounds and 1500 known inactive compounds were used for exploration, with the remaining compounds reserved for testing. This setting aims to verify exploration performance when available ligand data are relatively limited. We created 3 non-overlapping exploration sets and conducted independent explorations for each set.
(dataset-small): 10 known active compounds and 500 known inactive compounds were used for exploration, with the remaining compounds reserved for testing. This setting aims to verify exploration performance when available ligand data are limited. We created 3 non-overlapping exploration sets and conducted independent explorations for each set.

For each dataset, we create 3 pairs of exploration and test sets to evaluate generalization screening performance. The exploration and evaluation conducted for each pair are referred to as trial 1, 2, and 3. The 50-fold ratio of inactive compounds to active compounds reflects the design of DUD-E Generate Decoys, which produces about 50 decoys per active compound.

Note that for HIPK3, the number of known active compounds was insufficient, causing the numbers of ligands under the dataset-large and dataset-middle conditions to be nearly the same. Consequently, we use the dataset-large results as a reference for dataset-middle.

### 2.6 Evaluation of the proposed method

To evaluate the performance of our proposed method, we conducted the following experiments.

First, we assessed the screening performance using PDB structures. This allowed us to establish a baseline reference for screening performance against which subsequent results could be compared.

Next, to evaluate the screening performance using AlphaFold2 predicted structures, alanine substitutions were introduced into each column of the MSA with a probability of 0.5, and the average screening performance using the randomly generated structures was calculated. In addition, among the randomly generated predicted structures, we examined the screening performance that achieved the highest results on the test set. Through these two steps, we clarified the representative levels of screening performance using AlphaFold2 predictions can achieve.

We employed random search to explore alanine substitutions in the MSA, aiming to identify parameters that generate predicted structures more suitable for SBVS. As in the previous experiment, alanine substitutions were introduced into each column of the MSA with a probability of 0.5. Subsequently, we performed an exploration with a genetic algorithm to optimize alanine substitutions in the MSA.

Finally, we compared the screening performance of the predicted structures generated by our proposed method (via random search and a genetic algorithm) with that of the existing PDB structures and standard AlphaFold2 predictions. Through this comparison, we comprehensively evaluated the effectiveness of our proposed method and its advantages over alternative approaches.

For all experiments involving predicted structures (excluding those based on PDB structures), we generated and evaluated a total of 1100 predicted structures to ensure consistency in exploration and assessment.

## 3 Results

### 3.1 Comparison of methods based on the achieved screening performance

**Fig. 4:**
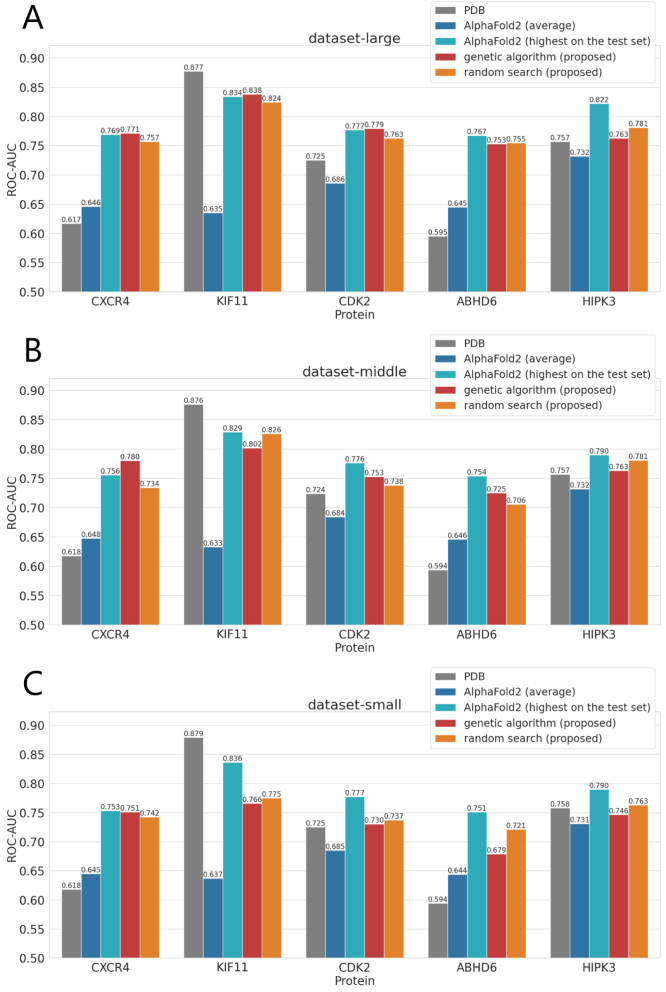
Comparison of screening performance (ROC–AUC) for predicted structures explored by the proposed methods (genetic algorithm and random search) with those of existing PDB structures and standard AlphaFold2 predictions. For all experiments involving predicted structures (excluding those based on PDB structures), 3 trials were conducted, and the average results were used for comparison. A, B, and C show the results for dataset-large, dataset-middle, and dataset-small, respectively.

Fig. 4 shows a comparison between the screening performance using the predicted structures explored by the genetic algorithm and random search, and the screening performance using PDB structures and AlphaFold2 predicted structures. First, in all cases, it was confirmed that the predicted structures explored by the proposed method (via random search and a genetic algorithm) were more suitable for SBVS than the average AlphaFold2 predicted structures. Furthermore, for four target (CXCR4, CDK2, ABHD6, and HIPK3), the predicted structures explored by the proposed method tended to be more appropriate for SBVS than the PDB structures. These results demonstrate that the proposed method outperforms conventional approaches in generating structures suitable for structure-based virtual screening. Notably, the four targets (CXCR4, CDK2, ABHD6, and HIPK3) are those for which the screening performance using PDB structures is relatively low. Therefore, it is considered that the proposed approach effectively enhances screening performance for such targets. Moreover, when comparing Fig. 4A, B, and C, it was observed that the screening performance using the explored predicted structures tended to improve as the number of ligands available for exploration increased. In particular, when a larger number of ligands was available for exploration, many cases were observed in which the predicted structures explored by the proposed method achieved test set ROC–AUC values that were comparable to or even exceeded the highest ROC–AUC observed among the AlphaFold2 predictions on the test set. Therefore, the proposed method is considered to be increasingly effective as the number of ligands available for exploration grows.

Comparing the proposed methods, random search and the genetic algorithm, it was observed that the greater the number of ligands available for exploration, the higher the tendency for the screening performance using the predicted structures explored by the genetic algorithm to exceed that achieved by random search. In dataset-large, for three targets with a high number of known active compounds in the exploration set (in descending order: CDK2, KIF11, and CXCR4), the genetic algorithm explored structures that outperformed those explored by random search. These findings suggest that the effectiveness of the genetic algorithm in exploring predicted structures suitable for SBVS increases with the number of known active compounds available. Conversely, when the number of ligands available for exploration is small, random search tended to yield predicted structures with higher screening performance than those explored by the genetic algorithm. In summary, when a sufficient number of known active compounds (30 or more) is available, exploration using the genetic algorithm is effective; however, when only a few active compounds are available, using random search is considered more appropriate.

### 3.2 Transition of exploration in a genetic algorithm

As illustrated in Fig. 3A, the ROC–AUC values for both the exploration and test sets increased over successive generations when sufficient active compounds were available. This indicates that the genetic algorithm effectively navigated the conformational search space to identify structures that generalized well to unseen compounds.

Additionally, even when temporary decreases in exploration ROC–AUC occurred, the algorithm eventually converged to a population enriched with high-performing MSA mutation patterns, enabling further refinement.

In contrast, for cases with limited active compound data (e.g., dataset-small), we observed that exploration performance did not always translate into test set gains. As shown in Fig. 3B, fluctuations and inconsistencies between exploration and test ROC–AUC curves emerged. This suggests that, under data-scarce conditions, the scoring signal becomes less reliable, potentially causing the genetic algorithm to stagnate or overfit to spurious patterns in the exploration set.

### 3.3 Distribution of the explored predicted structures

**Fig. 5:**
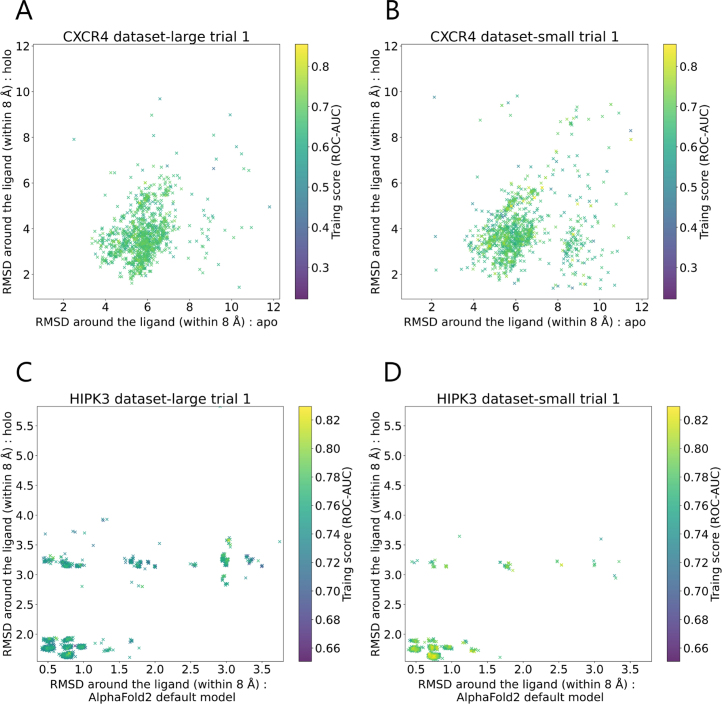
Examples of the distribution of explored AlphaFold2 predicted structures. Caption A shows trial 1 results for CXCR4 (dataset-large). Caption B shows trial 1 results for CXCR4 (dataset-small). Caption C shows trial 1 results for HIPK3 (dataset-large). caption D shows trial 1 results for HIPK3 (dataset-small).

We calculated the RMSD between residues within 8 Å of the ligand in the explored predicted structures and those in the apo and holo PDB structures, then visualized their distribution in a two-dimensional plot. For ABHD6 and HIPK3, no apo structure is registered in the PDB; thus, we instead used the default AlphaFold2 predicted structures. An example of the resulting distribution is shown in Fig. 5.

It is known that in a standard run of AlphaFold2, the predicted structure exhibits a single conformation [37]; however, as shown in Fig. 5, the introduction of MSA mutations enables the generation of a broad conformational space in AlphaFold2 predictions. This suggests that MSA mutations are effective in enhancing the structural diversity of AlphaFold2 predicted structures. Additionally, when comparing Fig. 5A and B, and C and D, the conformational space of the predicted structures did not shrink even when fewer ligands were available for exploration, indicating that the range of conformations the method can explore is not limited by the number of ligands used.

### 3.4 Transition of MSA mutations

**Fig. 6:**
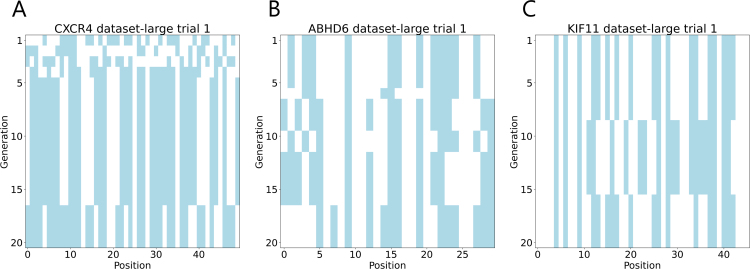
Examples of MSA mutation transitions. The vertical axis represents the generations of the genetic algorithm, while the horizontal axis shows the target residues for alanine substitutions arranged by residue number. White indicates no mutation, and blue indicates an alanine substitution.

We analyzed how the optimal MSA mutations changed in each attempt of the genetic algorithm. An example is depicted in Fig. 6. From Fig. 6, it can be seen that as the generations progress, mutations in specific residues gradually become fixed, indicating that the area around the previously discovered optimal MSA mutations continues to be explored. This observation implies that the genetic algorithm is functioning appropriately by refining its exploration for more suitable MSA mutations based on the best solutions identified so far.

### 3.5 Comparison of predicted structures explored by genetic algorithms and PDB structures

**Fig. 7:**
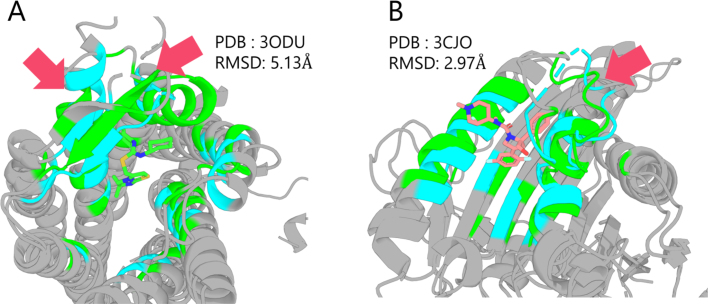
Examples of the comparison between predicted structures explored by a genetic algorithm and holo PDB structures. The predicted structure is shown in blue, and the PDB structure is shown in green. RMSD is calculated within 8 Å around the ligand.

We compared the tertiary structures of the best predicted structures obtained via the genetic algorithm with the holo PDB structures in order to evaluate which structural features influenced screening performance. We performed structural superposition in PyMOL, highlighting residues within 8 Å of the ligand as a binding site. The predicted structures used for comparison were those obtained in dataset-large. An example is shown in Fig. 7.

CXCR4 (Fig. 7A) exhibited a substantial improvement in ROC–AUC (0.617 to 0.793) relative to its PDB structure. A particularly large structural difference was observed in the helices surrounding the ligand, indicating that the search was able to explore configurations significantly different from those of the PDB structure.

For KIF11 (Fig. 7B), no improvement was observed relative to the PDB structure (0.877 to 0.849). The main difference appeared in a loop region highlighted by arrows. AlphaFold2 is reported to struggle with accurately predicting long loops of over 20 residues [38], and the loop in question spans 17 residues. Hence, it represents a region that AlphaFold2 inherently finds challenging to predict; although the exploration identified a conformation close to the PDB structure, this difficulty seems to have led to reduced ROC–AUC performance. Nevertheless, our method improved performance over the AlphaFold2 (highest on the test set) structure (ROC–AUC: 0.834), implying that the proposed method still yields benefits for targets that are difficult for standard AlphaFold2 predictions.

### 3.6 Applicability of the proposed method to novel target

As shown in Fig. 4, although ABHD6 and HIPK3 were not included among the training targets for AlphaFold2, AlphaFold2 was still able to generate predicted structures that achieved high screening performance for these proteins. This confirms the effectiveness of AlphaFold2 predicted structures in SBVS for novel targets.

Furthermore, for ABHD6 and HIPK3, the proposed method was able to explore structures that are more suitable than both the PDB structures and the average AlphaFold2 predicted structures. This highlights the applicability of the proposed method for novel targets.

## 4 Discussion

### 4.1 Comparison with AlphaFold3

AlphaFold3 [39], released by DeepMind in 2024, is the successor to AlphaFold2. One of its notable features is its ability to predict protein–ligand complexes. Given that holo structures tend to be more suitable for SBVS, AlphaFold3 has the potential to readily generate structures well-suited for screening. Accordingly, we evaluated the predictive performance of protein–ligand complex structures generated by AlphaFold3 under straightforward conditions and compared the results to those obtained by our proposed method.

**Fig. 8:**
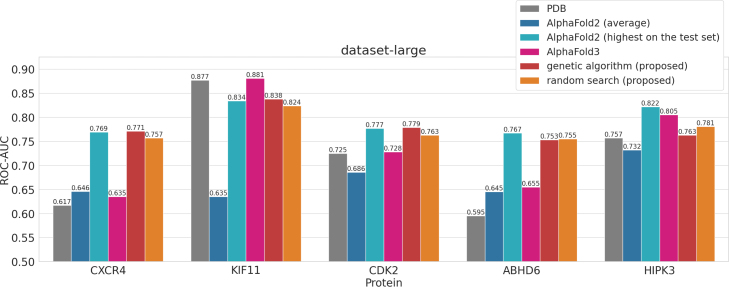
Comparison of methods based on the achieved screening performance (ROC–AUC), including AlphaFold3. The dataset-large results are shown.

The experimental procedure was as follows. First, we selected 10 representative ligands from the set of known active compounds by clustering. Next, for each representative compound, we ran AlphaFold3 five times and selected the structure with the highest average pLDDT. We then conducted docking simulations on these predicted structures and evaluated their screening performance. The results for dataset-large are shown in Fig. 8. Notably, AlphaFold3 was evaluated on a total of 10 structures, whereas the other methods were evaluated on 1100 structures.

From the results, it is apparent that the screening performance using AlphaFold3-predicted structures varies significantly depending on the target. Specifically, for KIF11 and HIPK3, AlphaFold3 tended to yield structures outperforming other methods. Conversely, for CXCR4, CDK2, and ABHD6, its performance was markedly lower. A notable trend is that KIF11 and HIPK3 are targets for which screening performance using PDB structures is already high, whereas the others are not. This suggests that AlphaFold3’s complex prediction capabilities may depend heavily on PDB-like structural patterns. In contrast, our proposed method, which emphasizes conformational exploration, might be more advantageous in low-PDB-performance targets.

### 4.2 Applicability and limitations

Our method assumes the availability of at least some ligand-binding information, and its applicability can be categorized into the following three scenarios:

• **Targets with experimental holo structures:** All 102 targets in the DUD-E dataset fall into this category. In such cases, our method aims to match or occasionally outperform PDB-derived holo structures through AlphaFold2-based structural modification. Improvements may arise due to enhanced conformational adaptability or better alignment with active ligands.
• **Targets with ligands but no holo structures:** Here, the main challenge is accurate binding site identification. Tools such as Fpocket or DynamicBind may assist, but misidentification can lead to misleading docking results. Nonetheless, poor discrimination between actives and inactives during screening may serve as a proxy indicator of such failure.
• **Targets with neither ligands nor holo structures:** Our method is not applicable in this case, as it requires active/inactive ligand data to guide conformational exploration. Without such data, docking-based optimization becomes unfeasible. Exploring ligand-agnostic scoring metrics or unsupervised structure selection strategies remains a future challenge.

### 4.3 Future directions

To enhance the scalability and robustness of our approach, several avenues are worth exploring. First, both AlphaFold2-based structure prediction and docking-based optimization are computationally intensive, and developing a more efficient pipeline is crucial for broader applicability. Second, we observed that exploration occasionally leads to increasing internal scores without corresponding improvements in actual screening performance. This suggests a need for more robust scoring functions, potentially incorporating ligand-specific or ML-based models. Finally, AlphaFold3 presents new opportunities for direct prediction of protein–ligand complexes. A hybrid approach that leverages AlphaFold3 for initial complex generation and our method for structural refinement could offer synergistic benefits.

## 5 Conclusion

In this study, we aimed to develop a methodology for achieving high-precision and versatile SBVS by leveraging AlphaFold2 predicted structures. Specifically, we evaluated the impact of various alanine substitution patterns in the MSA on the AlphaFold2 predicted structures using docking simulation scores with exploration compounds. Based on these results, we explored for alanine substitution patterns that can generate predicted structures suitable for SBVS—structures that enable the accurate evaluation of novel compounds. For the score-based exploration methods, both random search and a genetic algorithm were examined.

The results confirmed that, in all cases, the predicted structures explored via the proposed method were more suitable for SBVS than the average AlphaFold2 predicted structures. Furthermore, for the four targets CXCR4, CDK2, ABHD6, and HIPK3, the predicted structures explored via the proposed method tended to be more suitable for SBVS than the PDB structures. In addition, the screening performance using the explored predicted structures tended to be higher as the number of ligands available for exploration increased. In the comparison between the proposed methods, when a sufficient number of known active compounds (30 or more) was available, exploration using the genetic algorithm proved to be effective; conversely, when there were few active compounds, it appears more appropriate to employ random search. Moreover, for ABHD6 and HIPK3 – which are not included among AlphaFold2’s training targets – the proposed method was able to explore structures that were more suitable for SBVS than both the PDB structures and the average AlphaFold2 predicted structures. These findings support the applicability of our method to previously unseen targets, highlighting its generalizability beyond AlphaFold2 training data.

## CRediT authorship contribution statement

**Keisuke Uchikawa:** Writing – original draft, Visualization, Validation, Software, Methodology, Investigation, Formal analysis, Data curation, Conceptualization. **Kairi Furui:** Writing – review & editing, Validation, Software, Resources, Methodology, Investigation, Formal analysis. **Masahito Ohue:** Writing – review & editing, Validation, Supervision, Project administration, Methodology, Investigation, Funding acquisition, Formal analysis, Conceptualization.

## Code availability

The program code used in this study has been made publicly available on GitHub. The repository includes scripts for MSA mutation, AlphaFold2 structure generation, docking simulations, and the genetic algorithm implementation used in the study. The code can be accessed at the following URL: https://github.com/ohuelab/AF2_exploration.

## Declaration of Generative AI and AI-assisted technologies in the writing process

No declarations were made.

## Funding

This study was financially supported by JST FOREST, Japan (JPMJFR216J), 10.13039/501100001691JSPS KAKENHI, Japan (JP23H04880, JP23H04887, JP22K12258, JP23K28186), and AMED BINDS, Japan (JP25ama121026).

## Declaration of competing interest

None declared.

## Data Availability

The program code used in this study has been made publicly available on GitHub, https://github.com/ohuelab/AF2_exploration.

## Appendix A Supplementary data

MMC S1 Supplementary details on AlphaFold2 settings, GA parameters, targets, and software used in virtual screening.
