# Supplementary material for: Leveraging AlphaFold2 structural space exploration for generating drug target structures in structure-based virtual screening
Source: Biochem Biophys Rep. 2025 Jul 11;43:102110. doi: 10.1016/j.bbrep.2025.102110 (PMC12275061; doi:10.1016/j.bbrep.2025.102110)
Supplement: MMC S1 — Supplementary details on AlphaFold2 settings, GA parameters, targets, and software used in virtual screening. [file mmc1.pdf]

Supporting Information
Leveraging AlphaFold2 Structural Space Exploration for Generating Drug Target Structures in Structure-Based Virtual Screening

Keisuke Uchikawa[a], Kairi Furui[a], Masahito Ohue[a,*]

[a]*Department of Computer Science, School of Computing, Institute of Science Tokyo, 4259 Nagatsuta-cho, G3-56, Midori-ku, Yokohama, 226-8501, Kanagawa, Japan*

S1. AlphaFold2 Configuration

The following configuration of AlphaFold2 was used in this study:

1) --random_seed 1

2) --max_seq 16

3) --max_extra_seq 32

4) --num_recycle 1

5) --model_order 1

Furthermore, to ensure the reproducibility of the experimental results, the TensorFlow environment variable `TF_CUDNN_DETERMINISTIC` was set to 1 (`TF_CUDNN_DETERMINISTIC=1`), ensuring that identical inputs consistently produce identical outputs.

S2. Genetic Algorithm Configuration

Table S1 presents the settings of the genetic algorithm used in this study. Various settings used in this study were determined to be optimal based on preliminary experiments. The rationale for each configuration parameter is presented below.

- **Population Size: 50**
  The population size was set to 50 to ensure diversity within the algorithm while keeping computational costs manageable. This allows for adequate coverage of the search space.
- **Number of Generations: 20**
  The number of generations was set to 20 to balance computational cost and convergence. This setting ensures that computation time is appropriately controlled while achieving optimal results.
- **Mutation Rate: 0.05**
  The mutation rate was set to 0.05 to maintain diversity in the search while preventing performance degradation due to excessive mutations.

*Corresponding author

Table S1: Genetic algorithm configuration

Setting	Value
Population Size	50
Number of Generations	20
Mutation Rate	0.05
Crossover Probability	0.9
Mutation Method	bit-flip
Selection Method	elite preservation (2 individuals) +<br>tournament selection (tournament size = 5)
Crossover Method	two-point crossover
Scoring Method	ROC-AUC
Initial Population Generation Method	One individual generated without mutation and<br>149 individuals generated with a per-gene mutation probability of 0.5

- **Crossover Probability: 0.9**
  A high crossover probability of 0.9 was chosen to maximize the transfer of superior genes to the next generation while providing ample opportunities to generate new individuals.

- **Mutation Method: bit-flip**
  Bit-flip was adopted as the mutation method because directly switching genes between 1 and 0 allows for effective modification of genetic information.

- **Selection Method: elite preservation (2 individuals) + tournament selection**
  By preserving the top two elite individuals, this selection method guarantees that high-fitness individuals are retained in the next generation. Additionally, the use of tournament selection (with a tournament size of 5) helps maintain diversity while applying sufficient evolutionary pressure for convergence.

- **Crossover Method: two-point crossover**
  Two-point crossover was selected because it allows for the generation of diverse combinations between different parent individuals, thereby expanding the search space.

- **Initial Population Generation Method: One individual generated without mutation and 149 individuals generated with a per-gene mutation probability of 0.5**
  Given the short number of generations, the performance of the initial population significantly impacts the results. By increasing the number of initial individuals and including one individual without mutations, the method ensures diversity from the outset and enhances the stability of the search process.

S3. Information on the Targets Used

Table S2 presents the number of known active and inactive compounds utilized for each target, along with their corresponding PDB structure IDs. The apo PDB structures were obtained using Apo–Holo Juxtaposition (AHoJ) [1].

S4. Software Environment

The versions of the software utilized in this study are listed below.

- LocalColabFold (ColabFold) [2]: 1.5.5
- PyMOL Open-Source: 2.5.0

Table S2: Number of known active and inactive compounds and holo/apo PDB IDs for the targets

Target	#Actives	#Inactives	Holo PDB ID	Apo PDB ID
CXCR4	122	3,414	3ODU	4RWS
KIF11	197	6,912	3CJO	6TA4
CDK2	798	28,328	1H00	1W98
ABHD6	111	5,565	7OTS	-
HIPK3	43	2,450	7O7J	-

- Uni-Dock [3]: 1.1.1
- AutoDockTools: 1.5.7
- Open Babel: 3.1.1
- RDKit: 2023.09.6

References

[1] C. P. Feidakis, R. Krivak, D. Hoksza, M. Novotny, AHoJ: rapid, tailored search and retrieval of apo and holo protein structures for user-defined ligands, Bioinformatics 38 (24) (2022) 5452–5453.

[2] M. Mirdita, K. Schütze, Y. Moriwaki, L. Heo, S. Ovchinnikov, M. Steinegger, ColabFold: making protein folding accessible to all, Nature Methods 19 (6) (2022) 679–682.

[3] Y. Yu, C. Cai, J. Wang, Z. Bo, Z. Zhu, H. Zheng, Uni-dock: Gpu-accelerated docking enables ultralarge virtual screening, Journal of Chemical Theory and Computation 19 (11) (2023) 3336–3345.
